# Microstructure and Mechanical Properties of Weaving Wire and Arc Additive Manufactured AZ91 Magnesium Alloy Based on Cold Metal Transfer Technique

**DOI:** 10.3390/ma16114047

**Published:** 2023-05-29

**Authors:** Zhongrui Zhang, Junqi Shen, Ji Bi, Shengsun Hu, Yahui Zhen, Xianzheng Bu

**Affiliations:** 1 Tianjin Key Laboratory of Advanced Joining Technology, Tianjin University, Tianjin 300354, China; 2 School of Materials Science and Engineering, Tianjin University, Tianjin 300354, China; 3 International Institute for Innovative Design and Intelligent Manufacturing of Tianjin University, Shaoxing 312000, China; 4 Beijing Hangxing Machinery Co., Ltd., Beijing 100013, China

**Keywords:** AZ91 magnesium alloy, weaving wire and arc additive manufacturing, cold metal transfer, microstructure, mechanical property

## Abstract

Based on the cold metal transfer (CMT) technique, a deposited wall of AZ91 magnesium alloy was fabricated by weaving wire and arc additive manufacturing (WAAM), the shaping, microstructure, and mechanical properties of the sample with the weaving arc were characterized and discussed by compared with the sample without the weaving arc, and the effects of the weaving arc on grain refinement and property enhancement of the AZ91 component by CMT-WAAM process were investigated. After introducing the weaving arc, the effective rate of the deposited wall could be increased from 84.2% to 91.0%, and the temperature gradient of the molten pool could be reduced with an increase in constitutional undercooling. The equiaxed α-Mg grains became more equiaxial due to the dendrite remelting, and the β-Mg_17_Al_12_ phases distributed uniformly induced by the forced convection after introducing the weaving arc. Compared to the deposited component fabricated by the CMT-WAAM process without the weaving arc, the average ultimate tensile strength and elongation of the component by weaving the CMT-WAAM process both increased. The weaving CMT-WAAM component showed isotropy and has better performance than the traditional cast AZ91 alloy.

## 1. Introduction

The rapid expansion of metal use in recent years has caused a gradual depletion of non-renewable resources, severely limiting the advancement of human society, technology, and the economy. Due to their excellent mechanical properties, aluminum alloy and magnesium alloy have been used frequently as lightweight metals in a number of engineering fields [1,2]. Particularly, magnesium alloy has a wide range of application prospects in the automotive, military, aerospace, and biomedical industries due to its characteristics of high specific stiffness and strength, good thermal property, good damping property, and good electromagnetic interference resistance [2,3].

With a quick product cycle, wire arc additive manufacturing (WAAM) technology can be used to fabricate large and complex structural parts [4], and it has the potential to become one of the practical methods to manufacture high-performance magnesium alloy structures. At the moment, WAAM is used to fabricate a variety of alloys, including steel [5], aluminum alloy [6] and titanium alloy [7]. Due to the low density, low surface tension, high thermal conductivity, low melting and boiling points and large coefficient of linear expansion, magnesium alloy is susceptible to spattering, grain coarsening, porosity and thermal cracking during the traditional arc welding process [8,9,10]. As an improved gas metal arc welding (GMAW) process, the cold metal transfer (CMT) technique can achieve a spatter-free process with low heat input by the wire pullback and parameters control when the short-circuit occurs [11]. Therefore, CMT-based WAAM is gradually becoming a hotspot of magnesium alloy components manufacturing.

Inhomogeneous microstructure and anisotropic properties are common issues with magnesium WAAM components. Guo et al. [12] found that the microstructure of the deposited AZ80M wall by gas tungsten arc welding (GTAW) based WAAM varied greatly in different regions, and the properties of the specimen had obvious anisotropy. According to Yang et al. [13], the AZ31 deposited wall prepared by CMT-WAAM was primarily made up of columnar grains, and as a result, the sample’s tensile properties obviously showed anisotropy. Wang et al. [14] obtained a multi-layer single-bead sample of AZ31 magnesium alloy, and the results show that the mechanical properties are much higher than cast AZ31 magnesium alloy, but the columnar crystals grown along the deposited direction made significant anisotropy in tensile properties. Kiein et al. [15] prepared AZ61A single-bead specimen by using the CMT-WAAM, and they found that the specimens were mainly composed of fine equiaxed grain, but the weak fiber texture in it caused a slight anisotropy of mechanical properties. Therefore, in order to reduce the anisotropy of the microstructure and properties of magnesium WAAM components, it is necessary to introduce effective methods during the WAAM process. 

Many studies have shown that the properties of the prepared component can be improved by the refined grains, which can be realized by introducing external methods during the welding or WAAM process. Zhu et al. [16] found that the weaving arc was beneficial for the escape of gas from the molten pool during welding during the simulation of the GMAW process of 5083 aluminum alloy, thereby reducing the generation of pores. Mahajan et al. [17] introduced the weaving arc in the low-carbon steel GTAW process, which made the weld microstructure change from columnar grains to fine equiaxed grains, and the tensile strength of the weld area was improved. Biradar et al. [18] employed a transverse mechanical weaving arc to obtain grain refinement in the weldment during TIG welding of aluminum alloy, and it was found that fine-grained arc-weaved welds exhibited better yield and ultimate tensile strengths and significant improvement in percent elongation. Wei et al. [19] used a weaving arc in the fabrication of 2319 aluminum alloy samples by CMT-WAAM, and the results showed that the stirring of the weaving arc could influence the molten pool shape and improve the fluidity, changing the thermal distribution and the growth direction of columnar crystals; the weaving arc had an obvious effect on reducing the grain size and proportion of pores. Liu et al. [20] investigated the microstructure evolution and mechanical properties of GH4169 super alloy fabricated by WAAM with a weaving arc and compared it with the corresponding deposited samples; the results showed that the weaving arc could make smaller grains and fewer Laves phases, thus the sample had better uniform tensile properties and lower tensile anisotropies.

For magnesium alloy, introducing external methods during the process can also improve the properties of the prepared component. Cao et al. [21] found that the AZ31 magnesium alloy deposited wall with an equiaxed microstructure could be obtained by using ultrasonic frequency pulsed wire arc additive manufacturing (UFP-WAAM), which helped to achieve isotropic mechanical properties. According to Yuan et al. [22], the transverse weaving arc produced by an electromagnetic oscillator during the GTAW process of AZ31 and AZ91 magnesium alloys could significantly refine the grain structure of the welded metals. Subravel et al. [23] found that the magnetic weaving arc frequency could make the continual change in the weld pool shape, and thus the presence of finer grains and evenly distributed precipitates generated in the weld region of the joints. 

Although the good mechanical properties and isotropic characteristics of AZ91 magnesium alloy components fabricated by CMT-WAAM with a weaving arc had been confirmed by our previous study [24], the weaving effect and its mechanism had not been discussed yet. So, in this study, the deposited walls of AZ91 magnesium alloy were fabricated by using the CMT-WAAM with and without a weaving arc. The effects of the weaving arc on the macrostructure, microstructure and mechanical properties of the component were investigated systematically. Especially, the formation mechanisms of equiaxed grain and the reason for good mechanical properties induced by the weaving arc were discussed.

## 2. Experimental Procedure

The substrate used in the experiment was an as-cast AZ91 magnesium alloy plate with dimensions of 250 mm × 150 mm × 6 mm, and AZ91 wire with a diameter of 1.2 mm was selected as the filler material. Table 1 shows the chemical compositions of the substrate and filler wire. The deposition system was composed of a Motoman HP6 industrial robot (Yaskawa, Kitakyushu, Japan) and an Advanced 4000R welding machine equipped with an RCU 5000i controller (Fronius, Wels, Austria). 

**Figure 1:**
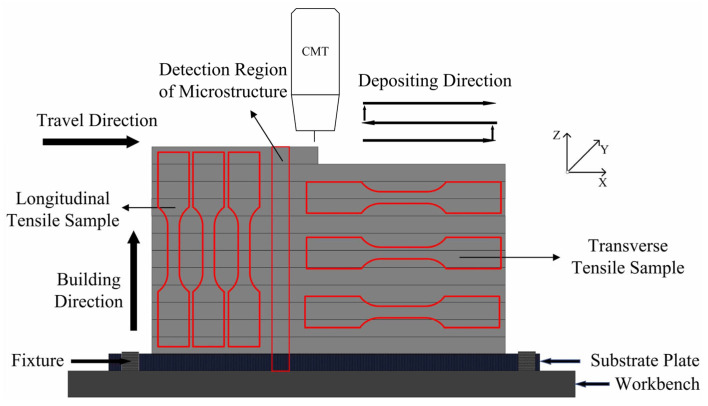
Schematic diagram of WAAM process and specimen preparation.

The process parameters of the deposition experience are referenced in trial experiments and our previous research [24]. The wire feed speed (WFS) and the traveling speed (TS) of the welding torch were kept at 12 m/min and 0.6 m/min, respectively. The flow rate of the shielding gas (i.e., pure argon) was 15 L/min. The substrate was cleaned to get rid of oxides and contaminants by using a grinder and then cleaned with alcohol to get rid of the organic substances before drying. The substrate was fixed on the workbenches after cleaning, to prevent deformation during the WAAM process. According to the results of the preliminary experiments and related studies [13,25], the reciprocating deposited path was selected to conduct the CMT-WAAM process in order to reduce the height difference between the two ends of the specimen, as shown in Figure 1. The cooling time between the adjacent layers was 3 min in order to reduce heat accumulation, which was helpful to prevent defects such as hot cracks and coarse grains. During this period, the surface of the deposited layer was cleaned to get rid of any new contaminants. The transverse length of the deposited specimen was at least 150 mm and the longitudinal height was at least 55 mm, which could provide enough area for cutting samples and tensile test specimens.

**Figure 2:**
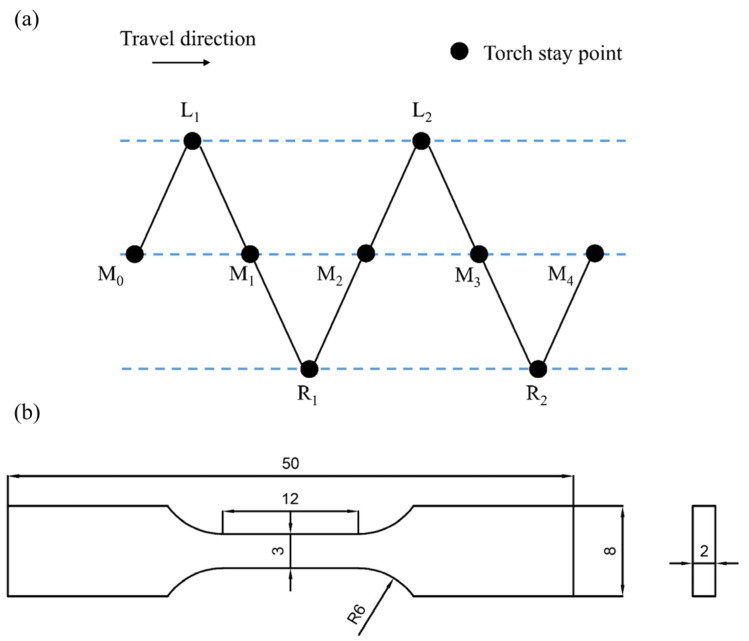
(**a**) Weaving path; (**b**) Dimensions of the tensile test specimens (unit: mm).

The introduction of the weaving arc was realized by using the ‘single oscillation’ weaving mode of the robot. As shown in Figure 2a, the torch moved along the path of M_0_-L_1_-M_1_-R_1_-M_2_, the points of M_i_, L_i_ and R_i_ represented the staying points of the middle, left and right, and in this study, the staying time of these staying points were set as 0.1 s, 0.2 s and 0.2 s, respectively. The weaving frequency was 5 Hz, and the weaving amplitude was 8 mm [24].

The samples cut from the specimens were polished according to the standard metallographic techniques and then etched in a solution of 10 mL HCl and 100 mL C_2_H_5_OH for 2 s [26]. An Axio Vert.A1 optical microscope (OM) (Carl Zeiss, Gottingen, Germany) was used to observe the microstructure of the specimen, and a D8 Advance X-ray diffraction (XRD) (Bruker, Karlsruhe, Germany) was applied to identify the phase compositions of the different areas in the deposited wall. The distribution and composition of the second phase were analyzed by using a JSM-7800F scanning electron microscope (SEM) (JEOL, Akishima, Japan) equipped with an energy dispersive spectroscope (EDS). An HV-1000A Vickers microhardness tester (HUAYIN, Laizhou, China) was used to measure the microhardness distribution of the deposited specimen with a load of 100 g and a holding time of 15 s. Three tensile specimens for each direction, i.e., longitudinal direction and transverse direction as shown in Figure 1, were cut from the deposited specimens, and they were tested by using an INSTRON 5848 electronic universal testing machine (Instron, Boston, America) with a loading rate of 2 mm/min by using static loading; the dimensions are shown in Figure 2b. The microstructure of the fractured surfaces was observed by SEM. 

The tensile strength anisotropy (*δ*_A_) of the deposited specimens was calculated according to Equation (1) [27]:

(1) $$\delta_{A}=\frac{\left(UTS_{\max}-UTS_{\min}\right)}{UTS_{\max}}\times 100\%$$

where *UTS*_max_ and *UTS*_min_ are the maximum and minimum values of the tensile strength in both directions.

## 3. Results and Discussion

### 3.1. Macroscopic Morphology

**Figure 3:**
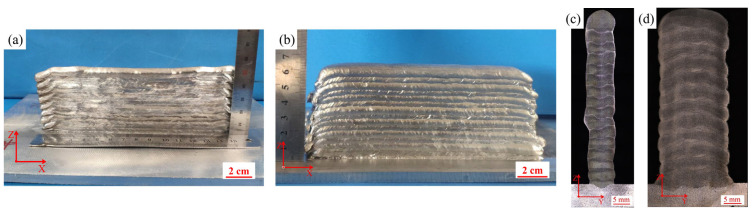
(**a**,**b**) Macroscopic morphology of the specimen by linear and weaving arc deposition; (**c**,**d**) Cross-sectional morphology of the specimen by linear and weaving arc deposition.

Figure 3a,b showed the macroscopic morphology of AZ91 deposited wall fabricated by CMT-WAAM with and without weaving arc. Both the deposited walls showed significant layering characteristics, with a small height difference between the two ends. Collapse and underflow phenomena were observed in the linear arc deposition specimen (i.e., without weaving arc), which were not obvious in the weaving arc deposition specimen. To sum up, the forming quality of both specimens was acceptable. As shown in Figure 3c,d, the specimens had no obvious defects such as pores and cracks, and there was little variation in the overall thickness of the deposited walls, indicating that the cooling time between the adjacent layers (i.e., 3 min) was conducive to the formation of the deposited wall. Unlike the linear deposition specimen, the fusion line of the weaving arc deposition specimen showed an obvious “M” shape, which was due to the 0.2 s and 0.1 s staying times on either side and middle of the weaving deposited bead.

**Figure 4:**
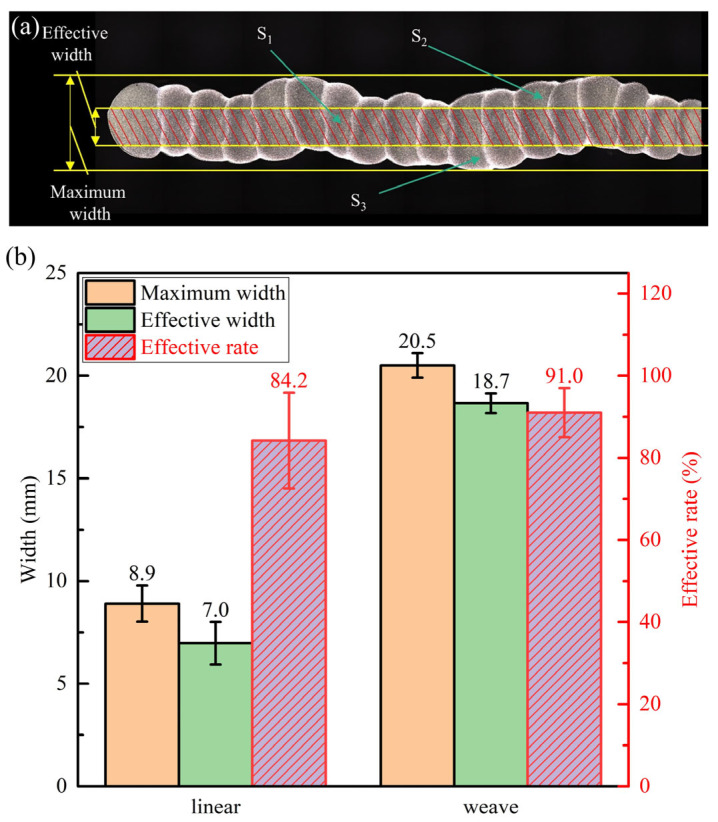
(**a**) Characterization of the deposited wall; (**b**) Forming characteristics of the specimens by different deposition process.

According to Figure 4a, the effective width, maximum width, and the area of *S*_1_, *S*_2_ and *S*_3_ of the deposited wall were measured by using the software Image J. The effective rate (*δ*_E_) of the deposited wall can be calculated by using Equation (2) [28]:

(2) $$\delta_{E}=\frac{S1}{S1+S2+S3}\times 100\%$$

where *S*_1_, *S*_2_ and *S*_3_ are the areas of the three regions shown in Figure 4a, respectively. *S*_2_ and *S*_3_ are the areas that will be removed by following the machining process. Therefore, a higher effective rate meant better utilization of the filler metal. As shown in Figure 4b, the effective width and effective rate of the specimen increased after the weaving arc was added from 7.0 mm and 84.2% to 18.7 mm and 91.0%, respectively. The weaving arc could improve effective width and effective rate by 167.1% and 8.1%. Compared with the increasing effective rate of 4.2% by optimizing process parameters [29], the weaving arc could increase the effective rate of the specimen obviously, which shows the same conclusion with Bultman et al. [30]. The increase in effective width and the effective rate was due to the wider deposited bead width caused by weaving arc deposition, which also made a flatter surface for the deposited bead, thus it could provide much support surface for the subsequent deposited bead.

### 3.2. Microstructure

**Figure 5:**
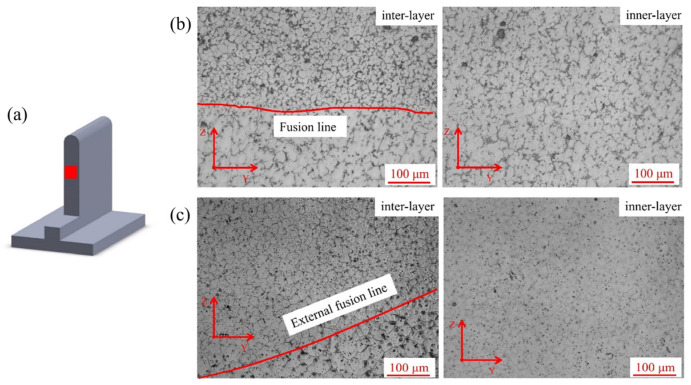
Microstructure of the specimens in the YZ plane: (**a**) Sampling location; (**b**) Microstructure of the linear arc specimen; (**c**) Microstructure of the weaving arc specimen.

**Figure 6:**
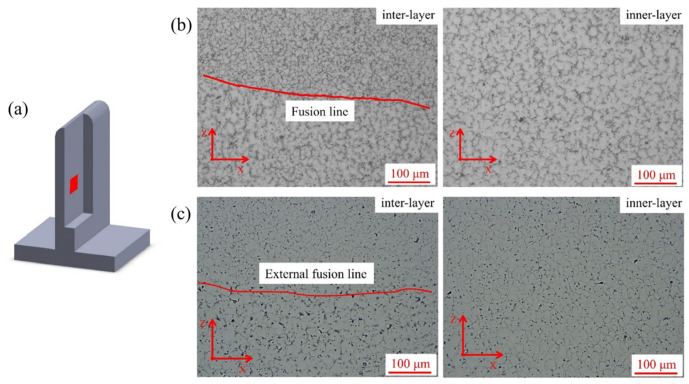
Microstructure of the specimens in the XZ plane: (**a**) Sampling location; (**b**) Microstructure of the linear arc specimen; (**c**) Microstructure of the weaving arc specimen.

**Figure 7:**
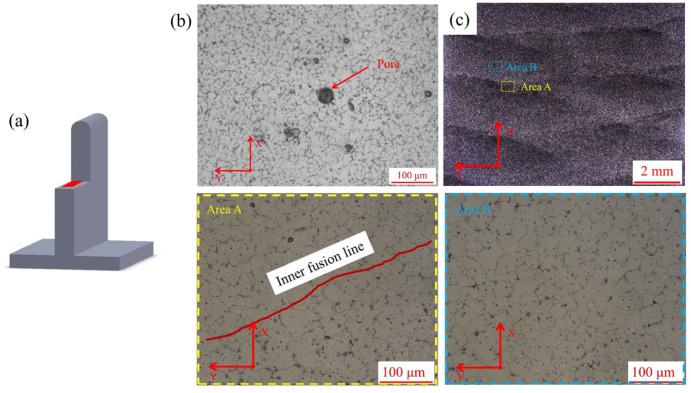
Microstructure of the specimens in the XY plane: (**a**) Sampling location; (**b**) Microstructure of the linear arc specimen; (**c**) Microstructure of the weaving arc specimen.

Figure 5, Figure 6 and Figure 7 showed the sampling locations and the corresponding microstructures of the deposited specimens in the YZ, XZ and XY planes, and there are obvious inner-layer and inter-layer regions. It can be found that both linear and weaving arc deposited specimens were all mainly composed of equiaxed grains. The grain aspect ratios of the linear and weaving arc specimens were 1.09 ± 0.02 and 0.98 ± 0.05, respectively, indicating that the grains of the deposited specimen became more equiaxial after introducing the weaving arc. In addition, the microstructure of the weaving arc specimen in the XY plane had a significant difference from that of the linear arc specimen. As shown in Figure 7c, the introduction of the weaving arc caused reciprocating fusion lines to form in the specimen along the XY plane, and there were two types of fusion lines that could be distinguished based on the characteristics of the fusion lines in the weaving arc deposited specimen. One was the “inter-layer fusion line” that forms between the deposited layers, and the other was the “inner-layer fusion line” that forms inside the same deposited layer due to the solidified metal being remelted by the reciprocating arc.

**Figure 8:**
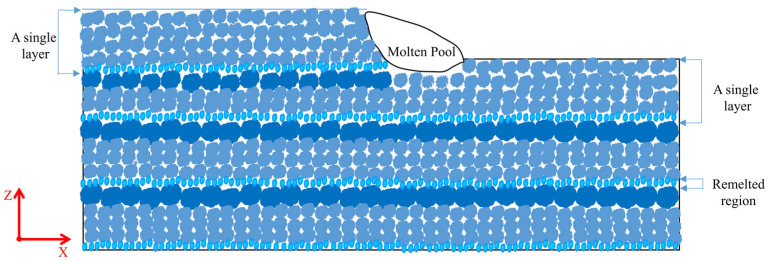
Microstructure formation process of the deposited wall.

Figure 8 showed the microstructure formation process of the deposited wall in the deposited direction. Due to the excellent heat dissipation conditions created by the bottom of the current deposited layer being in direct contact with the substrate or the previously deposited layer during the deposited process, fine equiaxed grains formed in the bottom of the deposited layer. However, the equiaxed grains in the upper and middle of the current deposited layer were relatively coarse, which was due to the great increase of the grains in the area nearby the molten pool when the top part of the previous layer was remelted as part of the molten pool. Eventually, the specimen was composed of equiaxed grains of different sizes. Additionally, as shown in Figure 7c, the grains near the inner-layer fusion line in the weaving arc specimen also showed a growing tendency, and this phenomenon was brought on by the weaving arc deposition process’ reheating of the solidified part of the deposited layer.

The preparation process, key metallurgical parameters, and the compositions and properties of the deposited materials all had an effect on the solidification structure of the deposited specimen. According to Wei et al. [31], the specimen’s final microstructure was determined by the temperature gradient *G* to grain growth rate *R* ratio, and when the ratio was large, columnar grains tend to be generated, resulting in the specimens’ tensile characteristics to be anisotropic. Compared with the WAAM, the selective laser melting (SLM) had a higher cooling rate and temperature gradient, which increased the *G/R*. As a result, the specimen prepared by SLM usually had tiny columnar grains while the specimen prepared by WAAM contained coarse equiaxed grains. Similarly, the AZ91 deposited wall in this study had larger grains than the specimen with the same material prepared by SLM [32]. 

The growth restriction effect could be induced by the constitutional undercooling at the solid-liquid interface during the solidification process, and the addition of Al could lead to grain refinement [33]. There was more Al element in AZ91 compared with AZ31, so greater component undercooling could be generated at the solid-liquid interface during the solidification process, which reduced the grain size [33]. StJohn et al. [34] proposed Equations (3) and (4) to determine the grain size of the alloy:

(3) $$Q=m_{l}c_{0}(k-1)$$

(4) d=a+b/Q

where *Q* is the growth restriction factor, *m_l_* is the slope of the liquidus line, *c*_0_ is the composition of the alloy, *k* is the partition coefficient, *d* is the typical grain size, *a* is the number of particles that actually nucleate grains at infinite values of *Q*, *b* is related to the potency of the nucleant particles, and under the same deposited condition, *a* and *b* are both constants. According to Equations (3) and (4), the growth restriction factor *Q* increased with the increasing Al content, thus restricting the growth of grains. Therefore, equiaxed grains were more likely to form in AZ91 than in AZ31 under the same conditions, and similar results were reported by Yuan et al. [22] and Paliwal et al. [35].

**Figure 9:**
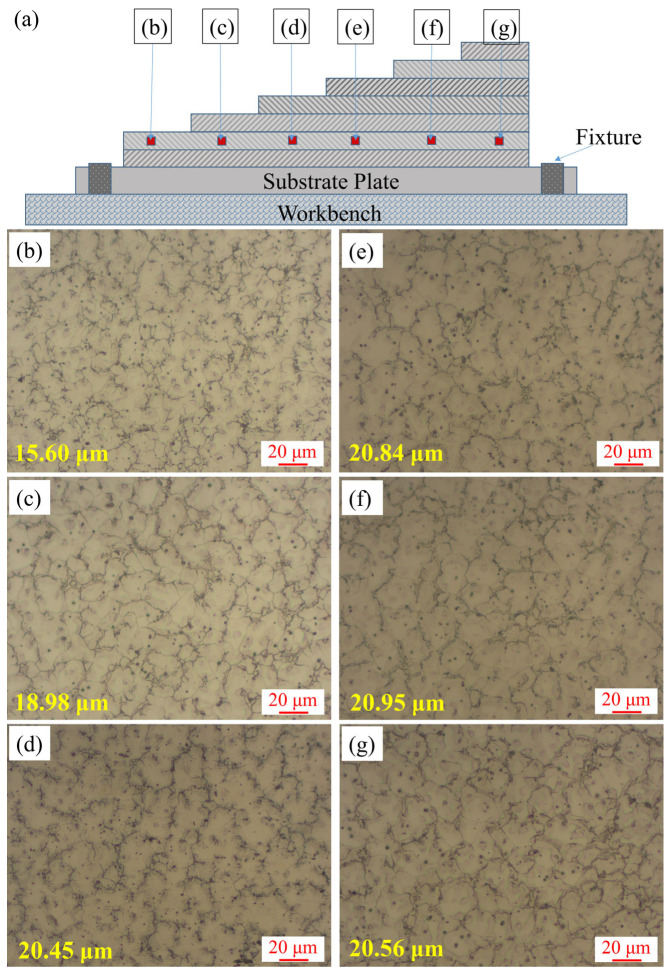
(**a**) Schematic of the stepped deposition test; (**b**–**g**) Microstructure of the corresponding zones in Figure 6a.

The previously deposited layer would be heated and put through some new thermal cycles when the following layers were deposited, so a stepped deposition test linear arc) was designed as shown in Figure 9a to determine the influence of the subsequent deposited layers on the microstructure of the previously deposited layer. Figure 9b–g showed the microstructure of the second layer after deposited different layers, the grains were all equiaxed and the average grain size of the second layer after depositing 2 to 7 layers were 15.60 μm, 18.98 μm, 20.45 μm, 20.84 μm, 20.95 μm and 20.56 μm, respectively. It could be found that the grains of the second layer coarsened after depositing three layers and four layer but remained stable throughout the deposition of layers 5 to 7. As a result, during the subsequent deposited processes, the grain size of the solidified layers increased, but it could no longer grow after more than two layers were deposited.

**Figure 10:**
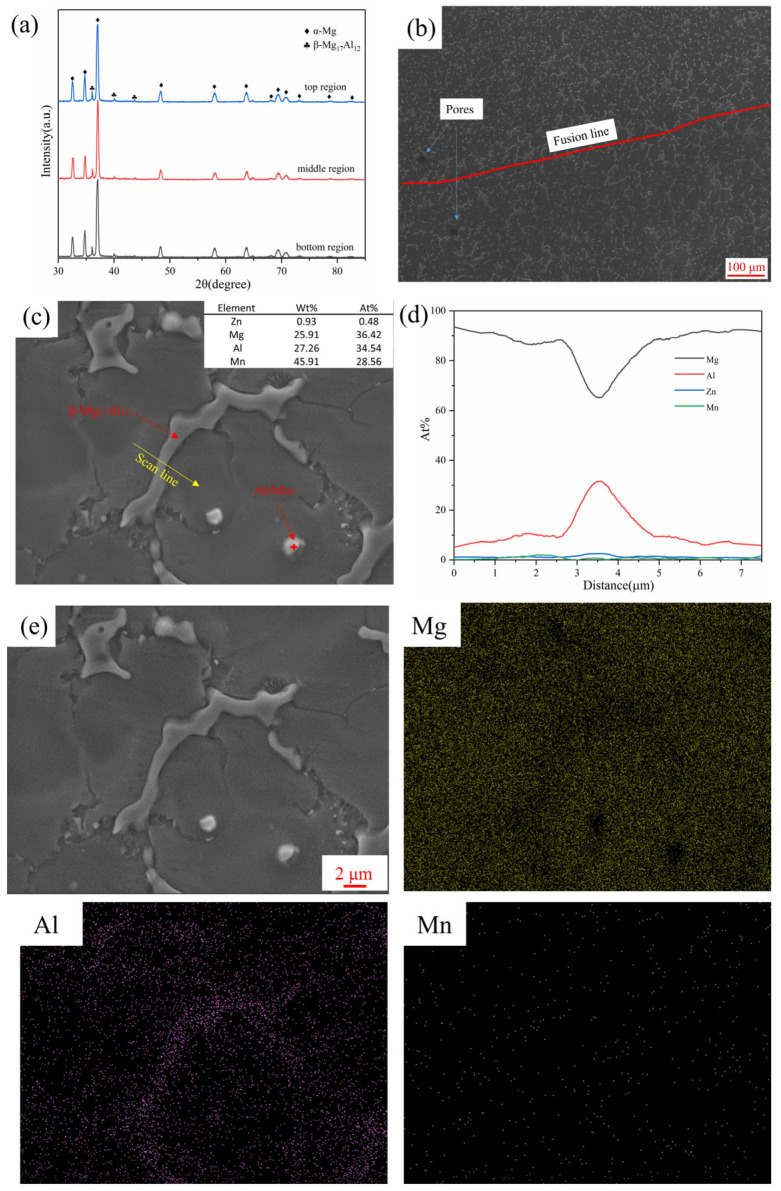
(**a**) XRD results of the linear arc deposited specimen; (**b**) Low magnification SEM image; (**c**) High magnification SEM image; (**d**) EDS line scanning result; (**e**) EDS mapping result.

Figure 10a showed the XRD results of different regions in the linear arc deposited specimen, which indicated that the specimens mainly consisted of α-Mg and β-Mg_17_Al_12_. As shown in Figure 10b, the stripe-like second phase was coarsened in the inter-layer region, but it still distributed along the grain boundary of the matrix α-Mg. Figure 10c,d showed the β-Mg_17_Al_12_ phases distributed along the grain boundaries of α-Mg in the inner-layer region, which was shaped strip-like and short rod-like. Additionally, a small amount of spherical secondary phase was found within the α-Mg and was identified as the Al_8_Mn_5_ phase according to the EDS results. Figure 10e illustrated that while there was some Al element in the α-Mg matrix, it was mainly concentrated in the β-Mg_17_Al_12_ phase. Due to the good thermal conductivity of the Mg alloy and the relatively high cooling rate, the Al element in the α-Mg matrix did not have enough time to diffuse to the grain boundaries during the CMT-WAAM process.

**Figure 11:**
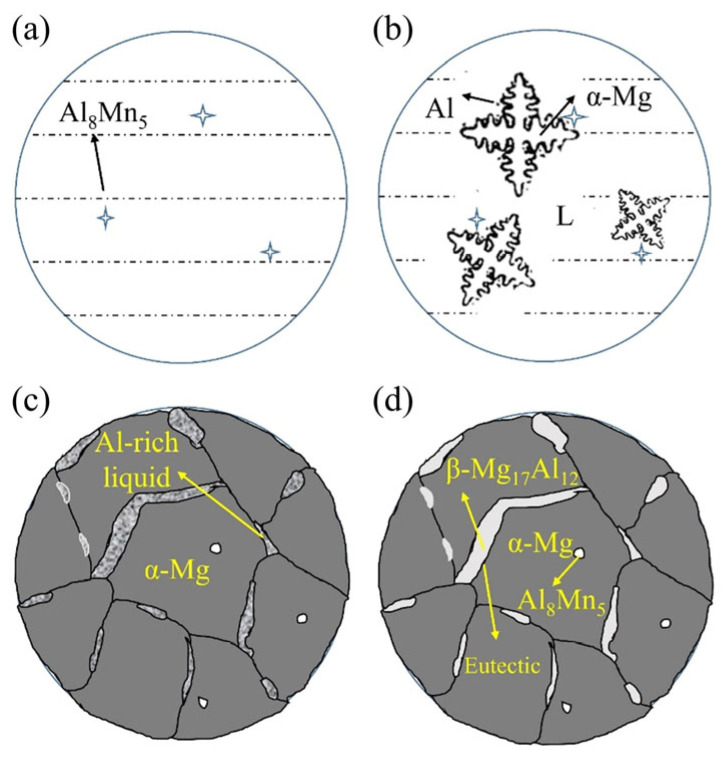
Solidification process of AZ91 alloy by linear arc deposition: (**a**) Al_8_Mn_5_ formation; (**b**) α-Mg formation; (**c**) β-Mg_17_Al_12_ formation; (**d**) Final microstructure.

As shown in Figure 11, some of the Al element was concentrated at the grain boundaries of the primary α-Mg as it nucleated and grew in a dendritic manner, resulting in the formation of β-Mg_17_Al_12_ second phase at the grain boundaries. The stripe-like β-Mg_17_Al_12_ phase eventually formed along the grain boundaries of α-Mg.

**Figure 12:**
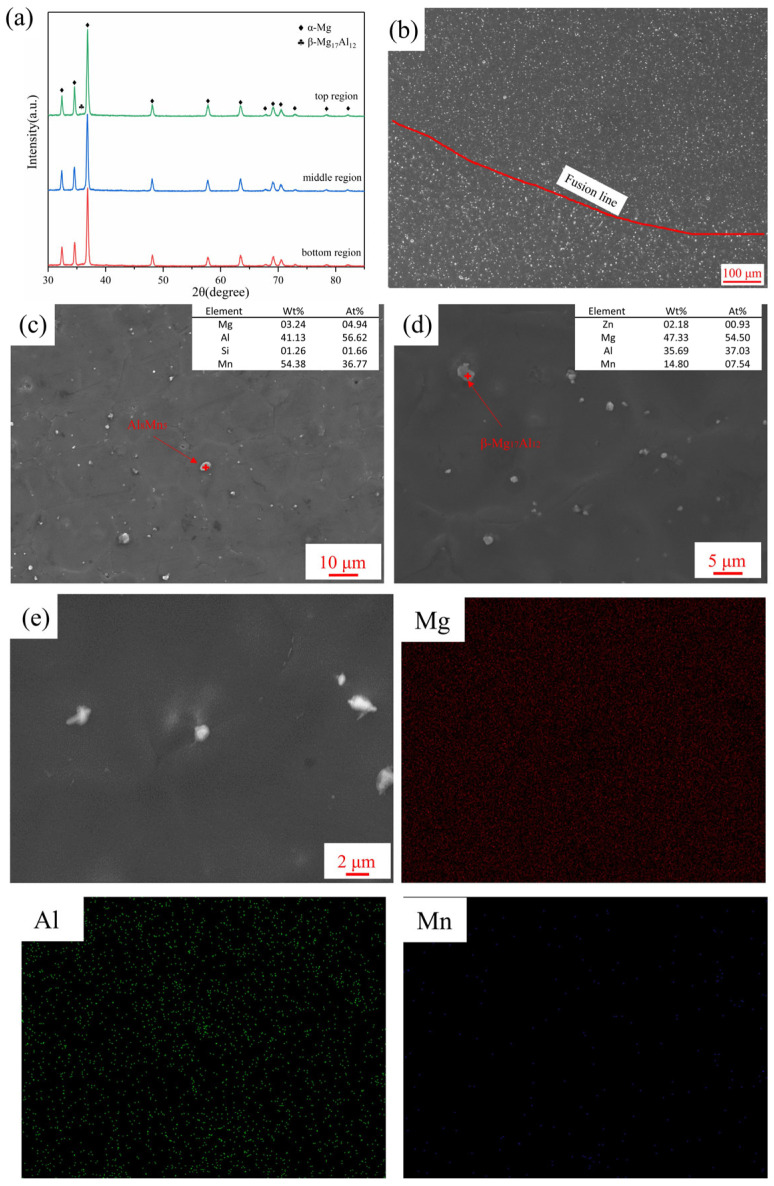
(**a**) XRD results of the weaving arc deposited specimen; (**b**) Low magnification SEM image; (**c**,**d**) High magnification SEM image; (**e**) EDS mapping result.

Figure 12a showed the XRD results of different regions in the weaving arc deposited specimen. As can be observed, the weaving arc did not change the growth orientation of the α-Mg phase but caused a significant decline in the diffraction peak intensity of the β-Mg_17_Al_12_ phase. As shown in Figure 12b, the β-Mg_17_Al_12_ was refined and distributed equally when compared with the linear arc deposited specimen. Although the β-Mg_17_Al_12_ phase had some coarsening below the fusion line, it still had a dispersive distribution. When the weaving arc was introduced, the volume fraction of the β-Mg_17_Al_12_ phase decreased from 11.51% to 1.47% (compared with Figure 10b and Figure 12b). Figure 12c,d indicated that the spherical second phase was distributed uniformly, and the specimen contained α-Mg, β-Mg_17_Al_12_ and Al_8_Mn_5_. According to the EDS result shown in Figure 12e, the distribution of Al elements was more uniform, and some Al elements were solid soluted in the α-Mg. As a result, the c/a-axis ratio of the α-Mg was reduced, and a non-basal slip could be activated at normal temperature, providing an additional slip system to improve the plastic deformation ability of AZ91 alloy at a normal temperature [36].

Natural convection and forced convection make up the majority of the liquid flow during the solidification of a molten pool. The natural flow of the molten pool is caused by the change in the local physical characteristics of the liquid metal, while the forced convection of the molten pool can be realized by using an electromagnetic field, mechanical vibration, and mechanical stirring. The flow of liquid metal can change the mass and heat transfer conditions in the molten pool as well as the grain growth process, thus affecting the microstructure morphology and composition segregation of the solidified metal [37]. Conduction and radiation are the two main ways of heat dissipation for the molten pool under the conditions of natural convection. Convection can also be used to achieve the heat dissipation of the molten pool when forced convection is present. By accelerating the molten pool flow, the weaving arc can alter the temperature distribution and gradient of the molten pool. The molten pool flow, meanwhile, can accelerate the solute atom transit and improve solute transport capacity, resulting in a more uniform composition of the solidified metal.

Relevant studies show that the introduction of a weaving arc can reduce the temperature gradient of the molten pool [22,38]. On the one hand, the weaving arc can force the liquid metal to flow, and then the temperature distribution of liquid metal becomes more uniform. However, the liquid stage is the only one where the forced convection works. On the other hand, the preheating action caused by the weaving arc is a significant factor in the decrease in the temperature gradient of the molten pool. The most remarkable feature of the weaving arc deposition process stands out for its periodic repeated heating compared with the linear arc deposition process. As shown in Figure 2a, the temperature from L_1_ to R_1_ is still rather high as the arc moves to point L_2_. As a result, the deposition process takes place under a preheating condition as the arc moves from L_2_ to R_2_. The three-dimensional heat conduction equation proposed by Rosenthal is as follows [39]:

(5) $$\frac{2\pi \left(T-T_{0}\right)kR}{Q}=\exp\left[\frac{-V\left(R-x\right)}{2\alpha }\right]$$

where *T* is the temperature (°C), *T_0_* is the preheating temperature (°C), *k* is the thermal conductivity of the material (W·m^−1^·°C^−1^), *R* is the distance from the origin (m), i.e., (*x*² + *y*² + *z*²)^1/2^, *Q* is the heat transferred by the heat source (J), *V* is the traveling speed of the welding gun (m·s^−1^), and α is the thermal diffusivity of the material (m^2^·s^−1^).

For the temperature gradient along the X-axis, since *y* = *z* = 0 and *R* = *x*, Equation (5) can be simplified as follows:

(6) $$T-T_{0}=\frac{Q}{2\pi kx}$$

Then, the temperature gradient *G* can be calculated by taking the partial derivative of Equation (6) concerning *x* [40]:

(7) $$G=\left(\frac{\partial T}{\partial x}\right)=\frac{Q}{2\pi k}\frac{-1}{x^{2}}=-2\pi k\frac{{\left(T-T_{0}\right)}^{2}}{Q}$$

According to Equation (7), the temperature gradient *G* is directly proportional to (*T* − *T*_0_)²; therefore, the increase of *T*_0_ can result in a significant decrease in the temperature gradient. As a result, the weaving arc can reduce the temperature gradient of the molten pool.

**Figure 13:**
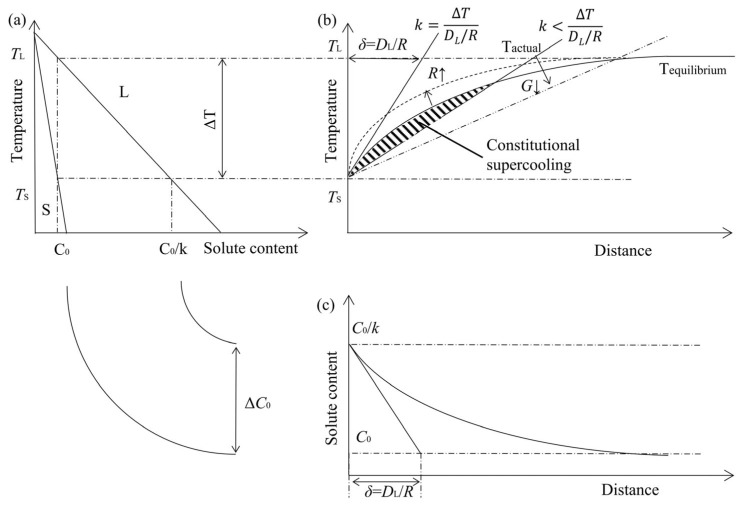
(**a**) Selected region of the Mg-Al equilibrium phase diagram; (**b**) Liquid-phase line temperature in front of the solid-liquid interface; (**c**) Liquid phase composition.

Part of the equilibrium phase diagram for the Mg-Al binary alloy (*k* < 1) is shown in Figure 13a,c shows the composition of the solute-rich boundary layer formed in front of the solid-liquid boundary and had a thickness of *D*_L_/*R*. Constitutional undercooling can emerge from the solute enrichment changing the temperature at which the liquid phase is near the interface. The relationship between temperature gradient *G* and growth rate *R* and the degree of undercooling is shown in Figure 13b [41]:

(8) $$G\leq \frac{\Delta T}{D_{L}/R}$$

The phenomenon of constitutional undercooling will appear if the slope of the tangent line of the liquidus at the solid-liquid interface and the relationship between the two obey Equation (8). The area of constitutional undercooling is indicated by the shaded area in Figure 13b. The discrimination of constitutional undercooling in the case of convection is based on the equation given below [42]:

(9) $$\frac{G}{R}<\frac{m_{L}C_{0}}{D_{L}\left(\frac{k}{1-k}\cdot e^{-\frac{R}{D_{L}}\delta }\right)}$$

where *m*_L_ is the slope of liquidus, and *D*_L_ is the solute diffusion coefficient in the liquid phase (m^2^·s^−1^).

The grain growth rate *R* can be increased to a certain level because the synthesized velocity of the weaving arc in both directions is greater than the linear deposition velocity. A significant degree of constitutional undercooling may result from the decrease in temperature gradient *G* or the increase in grain growth rate *R*, according to Equation (9) and Figure 13b. With an increase in the degree of constitutional undercooling, the microstructure morphology of the solidified metal will transform from plane grain to cellular grain to columnar grain, and finally to equiaxed grain.

As mentioned above, the introduction of a weaving arc could lead to forced convection. The dendrite arm may bend or even break when the shear stress caused by the forced convection exceeds the bending strength or shear strength of the dendrite arm at the solid-liquid interface. The fractured dendrite arm is then caught in the molten pool under the action of forced convection, thus being the core of heterogeneous nucleation to promote the formation of equiaxed grains. This mechanism is usually used in laser oscillation welding with relatively high frequency and scanning speed [43]. 

However, the forced convection caused by the weaving arc is limited, and the fluid shear stress it generates does not exceed that of the dendrite arm. Even yet, it can still cause stress concentration in the dendrite arms, thus promoting dendrite remelting. Additionally, forced convection can promote solute enrichment in the depression between the dendrite arms, which would lower the melting point there. The inner-layer fusion lines in Figure 7c provide evidence for the increase in temperature fluctuation near the solid-liquid interface caused by the reciprocating heating (induced by the weaving arc), which also causes the remelting of the dendrite arm.

According to the relevant research, the CMT technique can be used to add Ti powder as heterogeneous particles to prepare the aluminum alloy WAAM specimen with fine, equiaxed grains [44]. This is due to the fact that during the CMT-WAAM process, the high-temperature phases in the molten pool can serve as the heterogeneous particles for nucleation. However, the Mn content in AZ91 alloy is only 0.26%, and this content is insufficient to form enough Al_8_Mn_5_ phases with a sufficiently high precipitation temperature as the heterogeneous particles. 

In conclusion, the dendrite remelting brought on by the introduction of the weaving arc caused the grains of the deposited specimen to become more equiaxial.

The β-Mg_17_Al_12_ phase became finer with a dispersed distribution after introducing the weaving arc. There were two main reasons for this phenomenon, both of which were related to the nucleation and growth of the β-Mg_17_Al_12_ phase at the front of the solid-liquid boundary.

The forced convection caused by weaving arc could speed up the movement of solutes, heat, and atoms through the liquid phase, leading to an unorganized arrangement of atoms at the solid-liquid interface and fluctuation of the solid-liquid border. As a result, the roughness of the solid-liquid interface increased, which caused the nucleation rate of the second phase to increase. The number of depressions at the solid-liquid interface where Al atoms diffusing out of the α-Mg could be enriched due to the forced convection caused by the weaving arc, increasing the average undercooling of the solid-liquid interface and increasing the number of second phase nucleation sites. These fine second-phase nuclei were also captured by the interface before they gathered and grew, aiding in the formation of fine second-phase grains because the protrusions between the adjacent depressions might block or slow down the merging of these second-phase nuclei.

**Figure 14:**
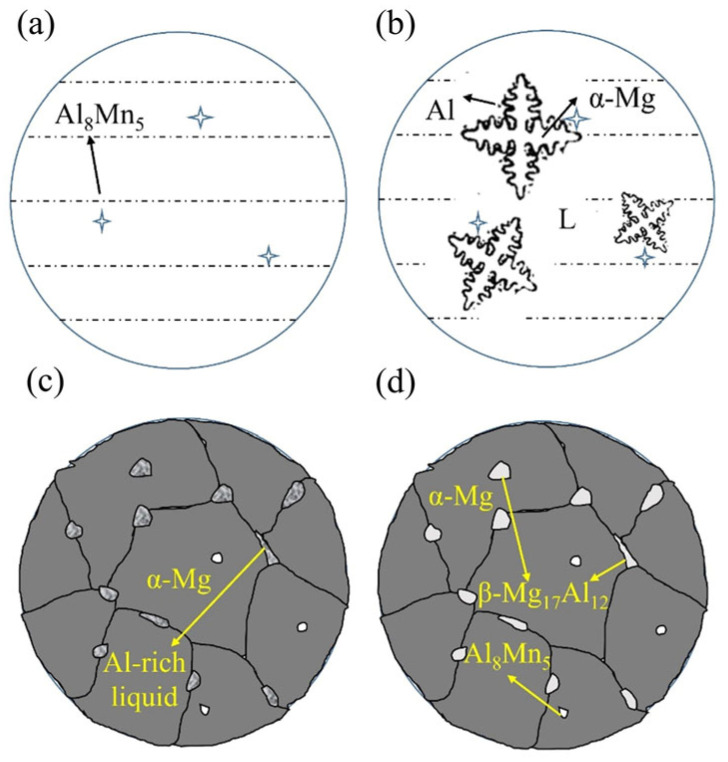
Solidification process of AZ91 alloy by weaving arc deposition: (**a**) Al_8_Mn_5_ formation; (**b**) α-Mg formation; (**c**) β-Mg_17_Al_12_ formation; (**d**) Final microstructure.

On the other hand, the large second-phase grains might become deformed or even fracture as a result of the forced convection of the liquid metal, and the Al atoms that were not enriched at the interface depressions were transported away by the flowing liquid metal. The second phase was unable to gather at the grain boundaries of α-Mg as a result. Figure 14 showed the solidification of AZ91 alloy during the weaving arc deposition process. Forced convection caused the β-Mg_17_Al_12_ phase to become fine and evenly dispersed, forming the microstructure in Figure 12b.

In conclusion, the introduction of the weaving arc lowered the temperature gradient of the molten pool and increase the undercooling, which promoted the formation of equiaxed grains. The equiaxed grains then became even more equiaxial due to dendrite remelting. The forced convection induced by the weaving arc could change the roughness of the solid-liquid interface, increase the nucleation rate of the β-Mg_17_Al_12_ phase, and decrease the aggregation and growth of the grain nucleus, thus refining the β-Mg_17_Al_12_ phase. Additionally, the flow of the β-Mg_17_Al_12_ phase could be accelerated by the forced convection, leading to a more even distribution.

### 3.3. Mechanical Properties

#### 3.3.1. Microhardness

**Figure 15:**
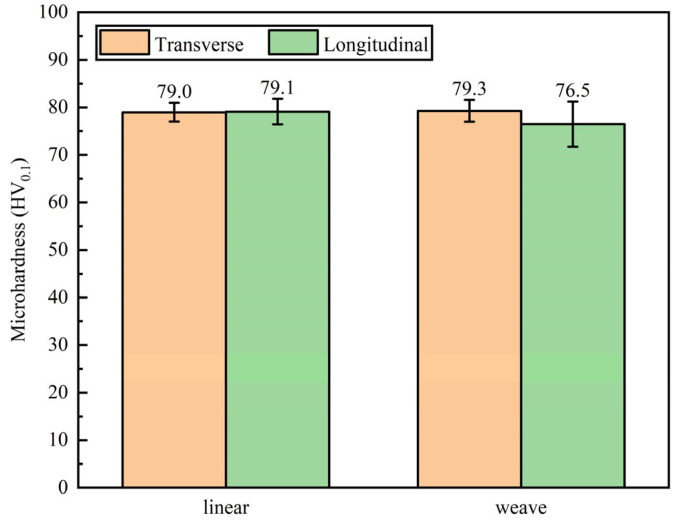
Microhardness distributions in vertical and horizontal directions by linear and weaving arc deposition.

Figure 15 showed the microhardness of the linear arc and weaving arc deposition specimen in both the transverse and longitudinal directions. The average microhardness in the two directions was 79.0 HV_0.1_ and 79.1 HV_0.1_, respectively, indicating the isotropy in the microhardness of the linear arc deposition specimen. The average microhardness of the weaving arc deposition specimen in the two directions was 79.3 HV_0.1_ and 76.5 HV_0.1_, respectively, which also showed isotropy in the microhardness. The isotropy in the microhardness showed the same conclusion with AZ91 GTAW-WAAM deposited wall but had a better performance than the 59.6 HV_0.2_ [45].

#### 3.3.2. Tensile Properties

**Figure 16:**
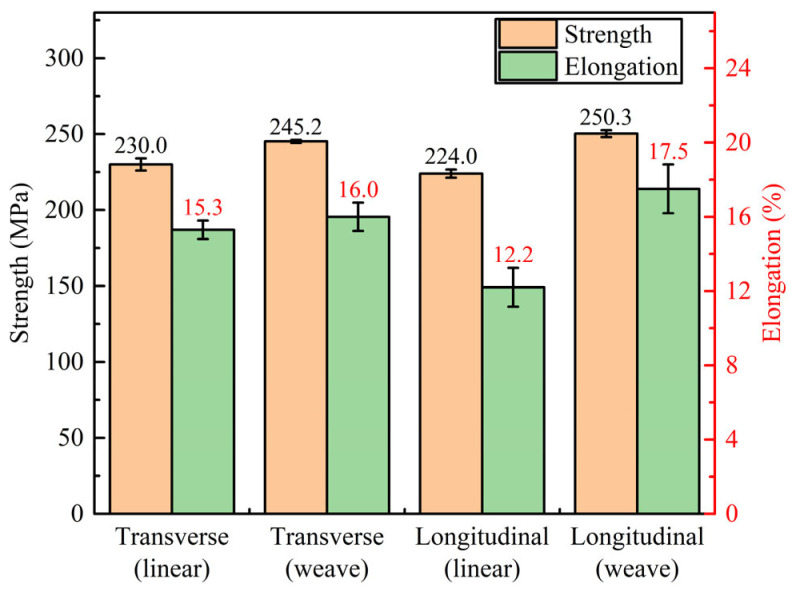
Tensile properties of the specimens by different deposition process.

Relevant research [12,13] indicated that the tensile properties of the deposited Mg-Al alloy specimens showed significant anisotropy between the direction transverse and longitudinal direction (anisotropy of 23.1% in [12] and 27.8% in [13]). However, in Figure 16 the average ultimate tensile strength (UTS) of the linear arc deposition specimen in the transverse and longitudinal directions was 230.0 MPa and 224.0 MPa, respectively, and those of the weaving arc deposition specimen were 245.2 MPa and 250.3 MPa, respectively. The maximum tensile strength anisotropy of the two specimens was 2.68% and 2.08%, which showed isotropy in UTS, coinciding with the conclusion of Cai et al. [45]. The average elongation (EL) of the linear arc deposition specimen in the transverse and longitudinal directions was 15.3% and 12.2%, respectively, and those of the weaving arc deposition specimen were increased to 16.0% and 17.5%, respectively. 

As shown in Table 2, after introducing the weaving arc, the UTS and elongation (EL) increased by 17.0% and 21.7% in average UTS and average EL based on the linear arc deposition specimen. The UTS of the weaving arc deposition specimen was similar to that of the GTAW-WAAM deposition specimen [45], a little higher than that of the diecast AZ91 magnesium alloy (230.0 MPa reported by Standard ASTM B94-18), and much higher than that of as-cast AZ91 magnesium alloy (124 MPa reported by the manufacturer of the substrate). In addition, the EL of both linear arc and weaving arc deposition specimens prepared by CMT was significantly higher than that of diecast and as-cast, which was related to the fine grains.

**Figure 17:**
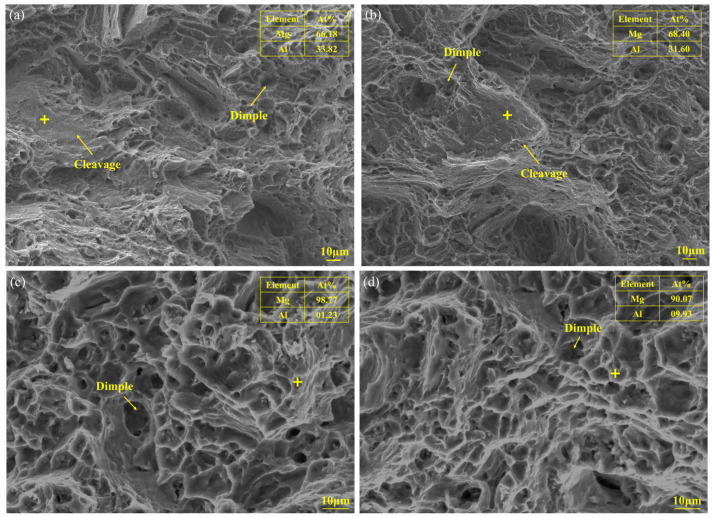
Fractured morphology of the tensile samples in transverse and longitudinal directions: (**a**,**b**) The linear arc deposition specimen; (**c**,**d**) The weaving arc deposition specimen.

Figure 17 showed the SEM fractured morphology of the tensile samples in transverse and longitudinal directions. The linear arc deposition specimen was composed of cleavages and dimples, which exhibited mixed fractures of ductile and brittle. Moreover, the weaving arc deposition specimen was mainly composed of dimples, showing typical ductile fracture. Compared with the linear arc deposition specimen, there are more dimples on the fracture surface of the weaving arc deposition specimen, which indicated that the weaving arc deposition specimen had better ductility, showing the same conclusion as the previous analysis of microstructure and UTS. The EDS results of the cleavage indicated that the failure originated from the β-Mg_17_Al_12_ phase, which formed along the grain boundaries of α-Mg. The EDS results of the dimple showed much more Mg element, indicating that the failure location was α-Mg.

**Figure 18:**
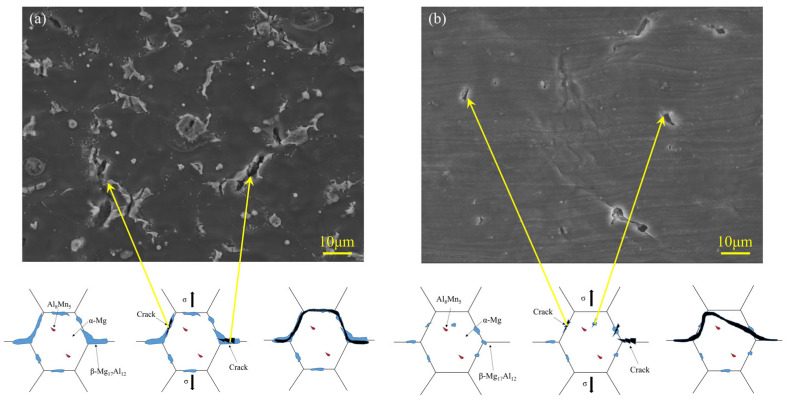
Microstructure of the tensile samples and the corresponding schematic of fracture process for: (**a**) Linear arc deposition specimen; (**b**) Weaving arc deposition specimen.

Figure 18 showed the tensile side of the fracture. For the linear arc deposition specimen, cracks initiated at the β-Mg_17_Al_12_ phases, which were distributed at the α-Mg grain boundaries during the tensile process, and then extended along the grain boundaries as the tensile stress increased, as shown in Figure 18a. This resulted in the intergranular fracture and cleavage formed. Figure 18b showed that cracks initiated at the fine β-Mg_17_Al_12_ phases in the weaving arc deposition specimen during the tensile process but were unable to extend along the grain boundaries due to the pinning effect caused by the fine β-Mg_17_Al_12_ phase. The cracks that did not easily expand along the grain boundaries resulted in the transgranular fracture and dimple formed.

## 4. Conclusions

Specimens of AZ91 magnesium alloy were fabricated by the CMT-WAAM technique, and the effect of weaving arc on the macroscopic morphology, microstructure evolution and mechanical properties of the specimen were analyzed. The main conclusions from this paper are as follows:

(1) Compared with the linear arc deposition specimen, the effective width and effective rate was increased from 7.0 mm and 84.2% to 18.7 mm and 91.0%, respectively, by introducing the weaving arc.
(2) The weaving arc deposition specimen was mainly composed of α-Mg grains and a fine β-Mg_17_Al_12_ phase spread fairly uniformly, as opposed to the linear arc deposition specimen, which included strip-like β-Mg_17_Al_12_ phase distributed at grain boundaries. The dendrite remelting caused the α-Mg grains more equiaxial when the weaving arc was introduced, the decrease in the temperature gradient of the molten pool and the increase in the degree of constitutional undercooling caused the β-Mg_17_Al_12_ phase to be refined and distributed uniformly.
(3) There was no obvious anisotropy in the mechanical properties of both specimens without and with weaving arc. The tensile properties were similar to those of the diecast AZ91 magnesium alloy and were significantly higher than the as-cast AZ91 magnesium alloy. The ultimate tensile strength and elongation of the weaving arc deposition specimen were 17.0% and 21.7% higher than that of the linear arc deposition specimen. In summary, the weaving arc had a certain increase in the mechanical properties of the deposited wall.
(4) The linear arc deposition specimen showed mixed fractures of ductile and brittle, while the weaving arc deposition specimen exhibited a distinguished ductile fracture.

## Figures and Tables

**Table 1:** Chemical compositions of the base metal plate and wire (wt. %).

Material	Al	Zn	Mn	Si	Fe	Cu	Ni	Mg
Substrate	8.70	0.58	0.24	0.02	0.002	0.005	0.001	Bal.
Wire	8.99	0.65	0.26	0.037	0.0018	0.0025	0.00043	Bal.

**Table 2:** Mechanical properties of AZ91 magnesium alloy prepared by different processes.

Material	Ultimate Tensile Strength (MPa)		Elongation (%)		Anisotropy (%)
	Transverse	Longitudinal	Transverse	Longitudinal	
GTAW-WAAM [45]	243.7	243.5	11.7	11.5	0.08
CMT-WAAM	230.0	224.0	15.3	12.2	2.68
CMT-WAAM by weaving arc	245.2	250.3	16.0	17.5	2.08
As-Cast (Substrate)	124.0		1.0		-
Die-Cast (Standard ASTM B94-18)	230.0		3.0		-

## Data Availability

The raw/processed data required to reproduce these findings cannot be shared at this time due to technical or time limitations.
